# Re-Audit of Documentation of Smoking and E-Cigarette Use History in Inpatient Medical Records on a Medium Secure Rehabilitation Ward

**DOI:** 10.1192/bjo.2026.11850

**Published:** 2026-06-30

**Authors:** Peter Robinson, Hamna Maryam, Amr Shehab, Neeti Sud

**Affiliations:** Cumbria Tyne and Wear NHS Foundation Trust, Newcastle upon Tyne, United Kingdom

## Abstract

**Aims:**

An audit of our 16 bedded male medium secure rehabilitation ward)during January - May 2025 identified that e-cigarette and smoking history was not recorded. Gap in availability of guidanceon how to take this history was identified.

We adapted standardised history and dependence checklists for our health setting for any new admissions. We then reaudited (August 2025 – December 2025) to assess if documentation of smoking and e-cigarette history had improved for any new admissions.

**Methods:**

We had 2 new admissions in the re-audit period. We also retrospectively interviewed the other 14 patients on the ward as per the action point from our original audit.Two of these patients did not have capacity to engage and one patient was away on leaves from the ward making interview impractical.

**Results:**

The use of the adapted interview ensured that 2 admissions in the reaudit period had a thorough smoking and vaping history documented. The average age in our cohort of 13 included patients including the new admissions was 41 years. Majority had a diagnosis of Schizophrenia.All patients had a history of smoking.

The average age of trying first cigarette was 13.3 years old with the majority starting smoking with friends. All but one patient used to smoke daily. The average age of starting smoking daily was 15 years old. Most had a family history of smoking and nine reported smoking inside the home. Retrospective assessment of smoking dependence using the Modified Version of the Fagerstrom Tolerance Questionnaire demonstrated that 5/12 had a moderate dependence and 7/13 had a substantial dependence. Three of the four patients who actively attempted to stop smoking were successful. Eight only stopped smoking when held in prison or admitted to hospital. The majority did not have concerns of smoking affecting their health at the time.

10/13 had used e-cigarettes with majority starting in hospital (5) or prison (3). Assessment of dependence using the E-cigarette Fagerström Test of Cigarette Dependence demonstrated that eight had a high dependence. One patient had successfully quit e-cigarette citing that motivation to quit was ‘flavours are horrible’ and used an inhalator. Two patients were independently reducing e-cigarette by reducing strength of liquid used, both citing health concerns for vaping. The majority (8/13) did not express health concerns from e-cigarette use.

**Conclusion:**

There is need for education of health impact of cigarettes /e-cigarettes and support for patients who are trying to quit e-cigarettes.

